# Analysis of Secretory Structures, Chemical Composition, and Anti-Inflammatory Properties of *Allophylus edulis* (A. St.-Hil., A. Juss. & Cambess.) Radlk Leaves

**DOI:** 10.3390/ph18101479

**Published:** 2025-10-01

**Authors:** Sidney Mariano dos Santos, Janaine Alberto Marangoni Faoro, Pedro Cruz de Oliveira Junior, Elisangela dos Santos, Candida Aparecida Leite Kassuya, Zefa Valdevina Pereira, Valter Paes de Almeida, Camila Dias Machado, Jane Manfron, Nadia Laiz Benites Souza, Claudia Andrea Lima Cardoso, Rosilda Mara Mussury, Anelise Samara Nazari Formagio

**Affiliations:** 1 Faculty of Health Sciences, Federal University of Grande Dourados, Dourados 79804-970, MS, Brazil; sidneysmariano@gmail.com (S.M.d.S.); janainefaoro@gmail.com (J.A.M.F.); elisangelaprocopiosan@gmail.com (E.d.S.); candida2005@gmail.com (C.A.L.K.); 2 Faculty of Biological and Environmental Sciences, Federal University of Grande Dourados, Dourados 79804-970, MS, Brazil; pedrojuniorbiologo@gmail.com (P.C.d.O.J.); zefapereira@ufgd.edu.br (Z.V.P.); nadialaissouza01@gmail.com (N.L.B.S.); maramussury@ufgd.edu.br (R.M.M.); 3 Postgraduate Program in Pharmaceutical Sciences, State University of Ponta Grossa, Ponta Grossa 84030-900, PR, Brazil; valterpaesdealmeida@gmail.com (V.P.d.A.); camiladiasmachado@hotmail.com (C.D.M.); jane@uepg.br (J.M.); 4 Center for Studies in Natural Resources, State University of Mato Grosso do Sul, Dourados 79804-970, MS, Brazil; claudia@uems.br

**Keywords:** vacum, leaf infusion, flavonoid, carrageenan, complete freund’s adjuvant, toxicity predictions

## Abstract

**Background/Objectives**: *Allophylus edulis*, known as “vacum”, is popularly used in Brazil for treating inflammatory diseases, though no scientific evidence supports the anti-inflammatory activity of its leaf infusion. This study aimed to assess the chemical composition, antioxidant and anti-inflammatory properties of the lyophilized infusion (ILAE) of *A. edulis* leaves, as well as the pharmacological effects of its hydromethanolic fraction (HMf) and the isolated compound vitexin 2″-O-rhamnoside (AE-1). Histochemical analyses of the leaves and in silico toxicity prediction of AE-1 were also performed. **Methods**: Fresh leaves were used for histochemical analysis and preparation of ILAE. The infusion was fractionated into *n*-hexane (Hf), ethyl acetate (EAf), and HMf fractions. Total phenols, flavonoids, flavonols, tannins, and antioxidant activity were determined by spectrophotometric methods. AE-1 was obtained from HMf through chromatographic methods and was evaluated by the ProTox model in relation to toxicity predictions (in silico). Anti-inflammatory effects of ILAE (3, 30, 100 mg/kg), HMf (3, 30 mg/kg), and AE-1 (3 mg/kg) were evaluated in carrageenan-induced paw edema, pleurisy, and CFA-induced inflammation in mice. **Results**: ILAE and its fractions were rich in total phenols (≤177 mg GAE/g) and showed potent antioxidant activity. Histochemical analysis revealed leaf secretory structures. AE-1 showed no hepatotoxic, carcinogenic, mutagenic, or cytotoxic effects in silico. All doses of ILAE and HMf reduced edema, hyperalgesia, and leukocyte migration. ILAE (30 mg/kg), HMf (30 mg/kg), and AE-1 (3 mg/kg) reduced CFA-induced inflammatory responses. **Conclusions**: ILAE contains polyphenolic compounds with antioxidant, anti-inflammatory, and antihyperalgesic properties, supporting the traditional use of *A. edulis* and its potential in inflammation-related therapies.

## 1. Introduction

The use of medicinal plants serves as the primary therapeutic approach for a wide range of diseases, especially within specific communities. Peruvian, Argentinian and Brazilian traditional communities report the popular use of leaves (aqueous maceration and/or infusion) from *Allophylus edulis* (A. St.-Hil., A. Juss. & Cambess.) Radlk. This plant (Sapindaceae family) is known as “vacum”, “cocu” or “chal-chal”, and is used for the treatment of several inflammatory conditions, including sore throat, stomach or liver pains, hepatitis, cold sores, hypertension, cholecystitis and prophylaxis [1,2,3,4,5].

Studies have provided evidence that extracts from the leaves (aqueous, ethanolic or methanolic) exhibit antimicrobial properties [6], angiotensin-converting enzyme inhibition activity [1], antioxidative effects [7], hepatoprotective potential [8], and negative ionotropic properties [9]. Additionally, chemical studies on the leaves of *A. edulis* (methanolic extract) have reported the presence of phenolic acids, coumarins and flavonoids in this species [1,8].

In the same context, our research group conducted studies that demonstrated anti-inflammatory activity in both the essential oil (volatile secondary metabolites, extracted by hydrodistillation) and its major components (non-polar). The anti-inflammatory potential was assessed through various methods, revealing significant inhibition of leukocyte migration, edema, cold sensitivity, and mechanical hyperalgesia induced by inflammatory and nociceptive agents in experimental models in mice [10,11,12]. However, to date, there is no scientific evidence demonstrating the anti-inflammatory properties of the popular herbal infusion prepared from *A. edulis* leaves, which primarily extract polar compounds. Aqueous or alcoholic extracts contain various polar compounds, including polyphenols, flavonoids, and alkaloids, and are obtained by standard extraction methods. Flavonoids from traditional medicinal plants such as hawthorn have shown broad pharmacological activities including redox modulation and anti-inflammatory responses [13]. Recent efforts to enhance the lipophilicity and oxidative stability of plant-derived phenolic compounds, such as resveratrol derivatives, highlight their potential in pharmaceutical development [14]. Quercetin and its analogs have demonstrated protective effects in inflammation-associated neuropathologies and kidney injuries, supporting the relevance of flavonoid-rich infusions [15,16].

To support the ethnobotanical use of *A. edulis* leaves for treating inflammation, this study evaluated the chemical composition, antioxidant and anti-inflammatory activities of the lyophilized infusion (ILAE), the hydromethanolic fraction (HMf) and the isolated compound vitexin 2″-O-rhamnoside (AE-1). Additionally, it included a detailed histochemical description and analysis of secretory structures of *A. edulis* leaves and in silico toxicity predictions of AE-1.

## 2. Results

### 2.1. Histochemical Analysis of Leaf Secretory Structures

**Figure 1:**
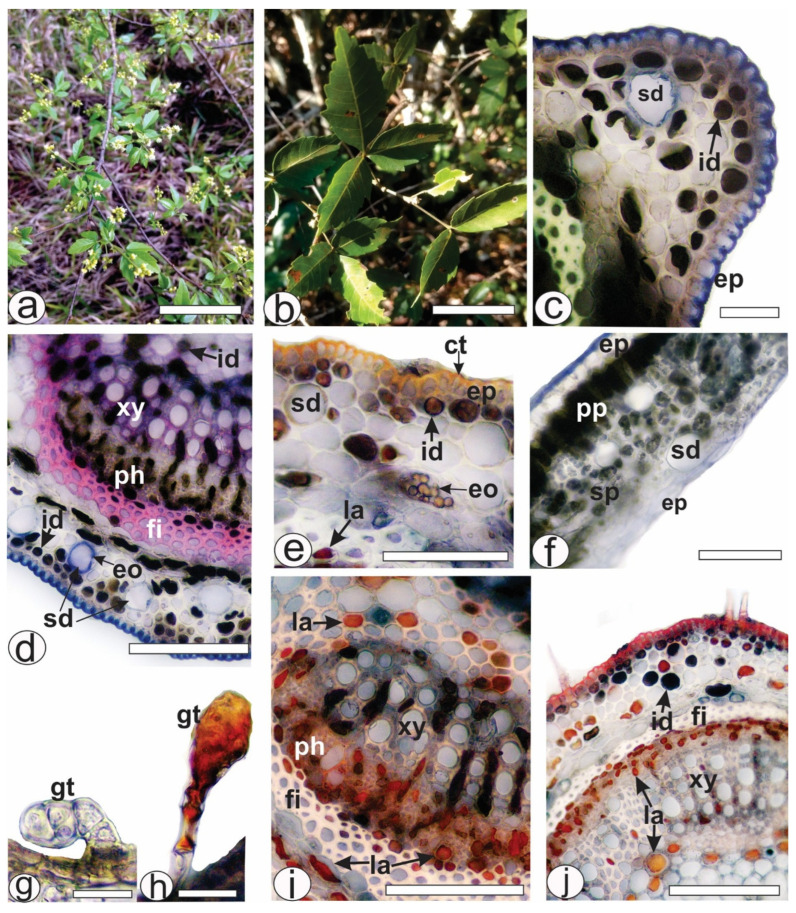
Histochemical analysis of leaf secretory structures of *A. edulis*. Fresh material. (**a**) Plant in inhabit. (**b**) A twig. (**c**,**d**) Detection of secretory ducts and essential oil by NADI reagent in the midrib. (**e**) Exposure of secretory ducts and essential oils by Sudan III in the midrib. (**f**) Positive result for secretory ducts in the lamina by NADI reagent. (**g**) Glandular trichome without reaction. (**h**) Observation of lipophilic material in the glandular trichome using Sudan III. (**i**) Detection of latex in the laticifers of petiole by red oil O reagent. (**j**) Positive result of the presence of latex in the laticifers of petiolule by red oil O reagent. [ct: cuticle, eo: essential oil, ep: epidermis, fi: fiber, gt: glandular trichome, id: idioblast, la: laticifer, ph: phloem, pp: palisade parenchyma, sd: secretory duct, sp: spongy parenchyma, xy: xylem]. Scale bars: (**a**) = 5 cm; (**b**) = 10 cm; (**d**,**e**,**f**,**i**,**j**) = 50 µm; (**c**,**g,**,**h**) = 20 µm.

**Figure 2:**
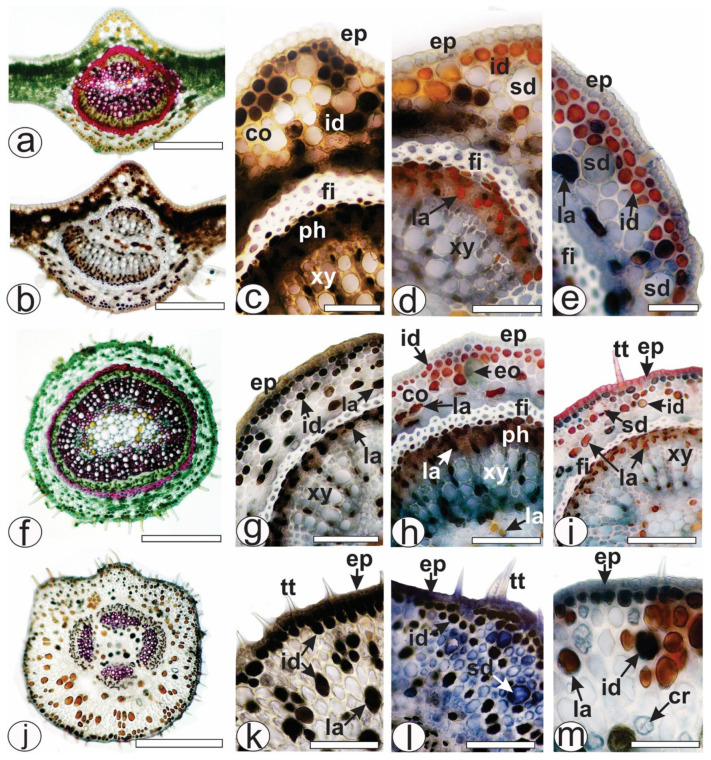
Histochemical analysis of leaf secretory structures of *A. edulis*. Fresh material. (**a**–**e**) Midrib. (**f**–**i**) Petiole. (**j**–**m**) Petiolule. (**a**,**f**,**j**) Positive reaction of fibers and xylem with phloroglucinol/HCl. (**b**,**c**,**k**) Detection of phenolic compounds by ferric chloride solution. (**d**,**h**) Detection of tannins by vanillin. (**e**) Detection of alkaloids in the laticifers by Dragendorff. (**g**) Phenolic compounds in reaction with potassium dichromate solution. (**i**,**m**) Detection of latex in the laticifers by red oil O reagent. (**l**) Detection of essential oils in the secretory ducts by NADI reagent. [co: collenchyma, cr: crystal, eo: essential oil, ep: epidermis, fi: fiber, id: idioblast, la: laticifer, ph: phloem, sd: secretory duct, tt: non-glandular trichome, xy: xylem]. Scale bars: (**f**,**j**) = 500 µm; (**a**,**b**) = 200 µm; (**c**,**g**–**i**,**k**,**l**,**m**) = 50 µm; (**d**,**e**) = 20 µm.

*Allophylus edulis* has compound leaves, trifoliate, with serrated margins and a pointed apex. The leaves (Figure 1a,b) showed four different types of secretory structures represented by ducts (Figure 1c–f and Figure 2d,e,i,l), glandular trichomes (Figure 1g,h), laticifers (Figure 1e,i,j and Figure 2g–i,k,m) and idioblasts (Figure 1c–e,j and Figure 2c,e,g–i,k–m). In the present study, secretory ducts were present in the midrib (Figure 1c–e and Figure 2a,b), lamina (Figure 1f), petiole (Figure 2f,i) and petiolule (Figure 2j,l). They were often found in or near the collenchyma (Figure 1c,d and Figure 2d,e). The secretion of the secretory ducts reacted positively with Sudan III (Figure 1e) and with NADI reagent, confirming in blue the presence of essential oil as secretion storage in ducts (Figure 1d). Glandular trichomes (Figure 1g,h) were rare and found on the epidermis of the leaves (midrib, lamina, petiole and petiolule). The secretion reacted with Sudan III, turning red-orange (Figure 1h) and blue in the NADI reaction, indicating the presence of essential oils.

Laticifers were observed in large numbers in the ground parenchyma of the midrib (Figure 1e and Figure 2d,e), petiole (Figure 1i and Figure 2h,i) and petiolule (Figure 1j and Figure 2m). Specifically, laticifers were present in the collenchyma in the ground parenchyma and near the fibers (Figure 1e,j and Figure 2g–i,k,m), in the phloem (Figure 1i,j and Figure 2d,g–i), near the xylem and in the pith (Figure 1i,j and Figure 2h). Latex reacted positively with red oil O (Figure 1i,j and Figure 2i,m) in the histochemical tests. Different chemical classes of metabolites were detected in the latex, such as lipids were detected with Sudan III (Figure 1e), phenolic compounds with ferric chloride (Figure 2c,k) and potassium dichromate (Figure 2g). Tannins were detected by vanillin only in the laticifers of phloem (Figure 2d,h) and alkaloids were distinguished by Dragendorff method (Figure 2e).

Secretory idioblasts were frequently observed in leaves of *A. edulis* (midrib, petiole and petiolule). They were found under the epidermis in rows (Figure 1c–e and Figure 2c–e), scattered in the collenchyma (Figure 1c–e,j and Figure 2c–e) and in the parenchyma cells of the xylem (Figure 1i) and in small amounts in the pith (Figure 1d). The histochemical tests showed the presence of lipids (Figure 1e) and phenolic compounds (Figure 2c,g,k) in the idioblasts. Tannins were detected by vanillin only in the idioblasts of the collenchyma (Figure 2d) but not found in the idioblasts present in the xylem (Figure 2h). Laticifers differed from idioblasts in cell diameter, shape, color, localization of storage, and the chemical composition of secretion (Figure 1e,i,j and Figure 2d–i).

### 2.2. Chemical Study

The ILAE and the fractions obtained by partitioning (EAf and HMf) exhibited moderate levels of total phenols ≤ 177 mg GAE/g, flavonoids ≤ 64 mg QE/g, flavonol ≤ 55 mg QE/g, and condensed tannins ≤ 44 mg CE/g, as shown in Table 1. These results are in agreement with the methods and calibration curves described.

**Figure 3:**
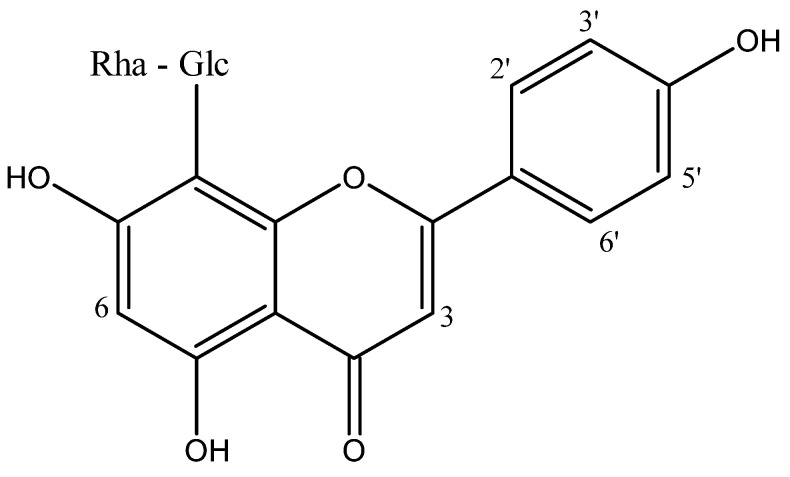
Chemical structure of vitexin 2″-O-rhamnoside (AE-1).

The partitioning of the HMf led to the isolation and identification of a flavone C-glycoside, vitexin 2″-O-rhamnoside (AE-1) (Figure 3). The presence of proton signals at H-6 (δ_H_ 6.58) and H-3 (δ_H_ 6.80), located in rings A and C, respectively, is consistent with a substitution at position C-8 of the apigenin skeleton. The ^1^H and ^13^C NMR spectra of AE-1 displayed characteristic signals for a ring B, consistent with a mono-oxygenated ring at C-4′, as indicated by ortho-coupled aromatic protons/carbon at δ_H_ 8.04 (d, *J* = 7.8 Hz)/δ_C_ 128.8 and δ_H_ 7.44 (d, *J* = 7.8 Hz)/δ_C_ 115.6. Additionally, the presence of sugar moieties at C-8, identified as glucose-2″-O-rhamnose was evidenced by the signals in δ_H_ 5.51 (d, *J* = 4.5 Hz)/δ_C_ 71.5, δ_H_ 5.47 (d, *J* = 1.8 Hz)/δ_C_ 100.5, and a series of multiplets for sugar protons between δ_H_ 4.39 and 3.03. The methyl group of rhamnose was observed at δ_H_ 1.04 (d, *J* = 6.0 Hz)/δ_C_ 17.6. The AE-1 was identified by comparing spectroscopic data (^1^H and ^13^C NMR) with data from the literature [17,18].

^1^H NMR δ_H_ (300 MHz, CD_3_OD): 7.44 (d, *J* = 7.8 Hz, H-2′/6′), 8.04 (d, *J* = 7.8 Hz, H-3′/5′), 6.80 (s, H-3), 6.58 (s, H-6), 5.51 (d, *J* = 4.5 Hz, H-1″ Glc), 5.47 (d, *J* = 1.8 Hz, H-1‴ Rha), 4.39–3.03 (m) sugar protons, 1.04 (d, *J* = 6.0 Hz, CH_3_). ^13^C NMR δc (75.5 MHz, CD_3_OD): 164.1 (C-2), 102.5 (C-3), 182.3 (C-4), 161.1 (C-5), 98.4 (C-6), 162.5 (C-7), 104.4 (C-8), 155.8 (C-9), 103.4 (C-10), 121.8 (C-1′), 115.6 (C-2′/6′), 128.8 (C-3′/5′), 160.8 (C-4′), 71.5 (C-1″ Glc), 74.8 (C-2″), 79.7 (C-3″), 70.5 (C-4″), 81.5 (C-5″), 61.2 (C-6″), 100.5 (C-1‴ Rha), 70.2 (C-2‴), 71.6 (C-3‴), 70.3 (C-4‴), 68.4 (C-5‴), 17.6 (CH_3_).

**Figure 4:**
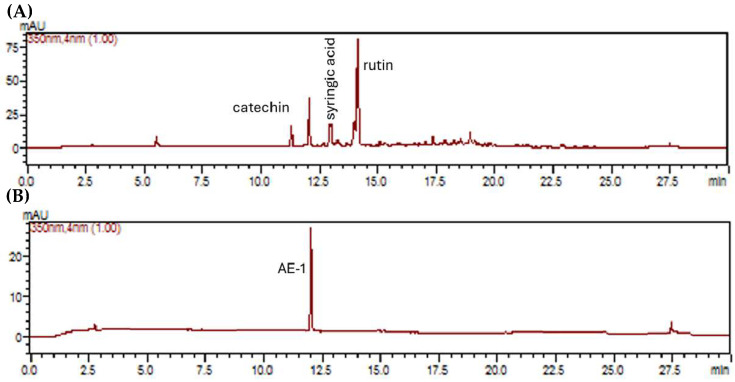
Representative LC-DAD chromatograms recorded at 350 nm of (**A**) ILAE and (**B**) AE-1.

The ILAE exhibited phenolic and flavonoid contents, and an LC-DAD method was developed to identify and quantify its components by comparison with authentic standards as catechin (rt = 11.26 min, 3.3, mg/g), syringic acid (rt = 12.95 min, 4.2 mg/g), rutin (rt = 14.29 min, 23.7 mg/g) and isolated compound AE-1 (rt = 12.45 min, 10.4 mg/g) (Figure 4A,B).

### 2.3. In Silico Toxicity Prediction of AE-1

The ProTox toxicity model showed that AE-1 is predicted to be free of hepatotoxicity or parameters related to carcinogenicity, mutagenicity, and cytotoxicity effects. However, the database indicated that AE-1 has an immunotoxic effect (Table 2). When submitted to the server of oral toxicity analysis in rodents in in silico model, it showed class 5 toxicity, with a predicted LD_50_ of 5000 mg/kg and an accuracy of 67.38% (Table 2).

### 2.4. Antioxidant Activity

The results showed that ILAE, EAf and HMf have potent antioxidant activity by reducing the DPPH radical (IC_50_ ≤ 28.07 µg/mL) and ABTS (IC_50_ ≤ 61.80 µg/mL), highlighting the HMf as the most potent fraction, with IC_50_ = 15.17 µg/mL and IC_50_ = 25.15 µg/mL), respectively (Table 3). Higher IC_50_ values (≤195.60 µg/mL) were observed for ILAE and EAf in the lipid peroxidation assay using the β-carotene/linoleic acid method. Once again, highlighting HMf as the most potent (IC_50_ of 55.44 µg/mL), when compared to BHT (IC_50_ = 13.03 µg/mL) (Table 3).

### 2.5. Anti-Inflammatory and Anti-Hyperalgesic Activity of ILAE, HMf and/or AE-1

**Figure 5:**
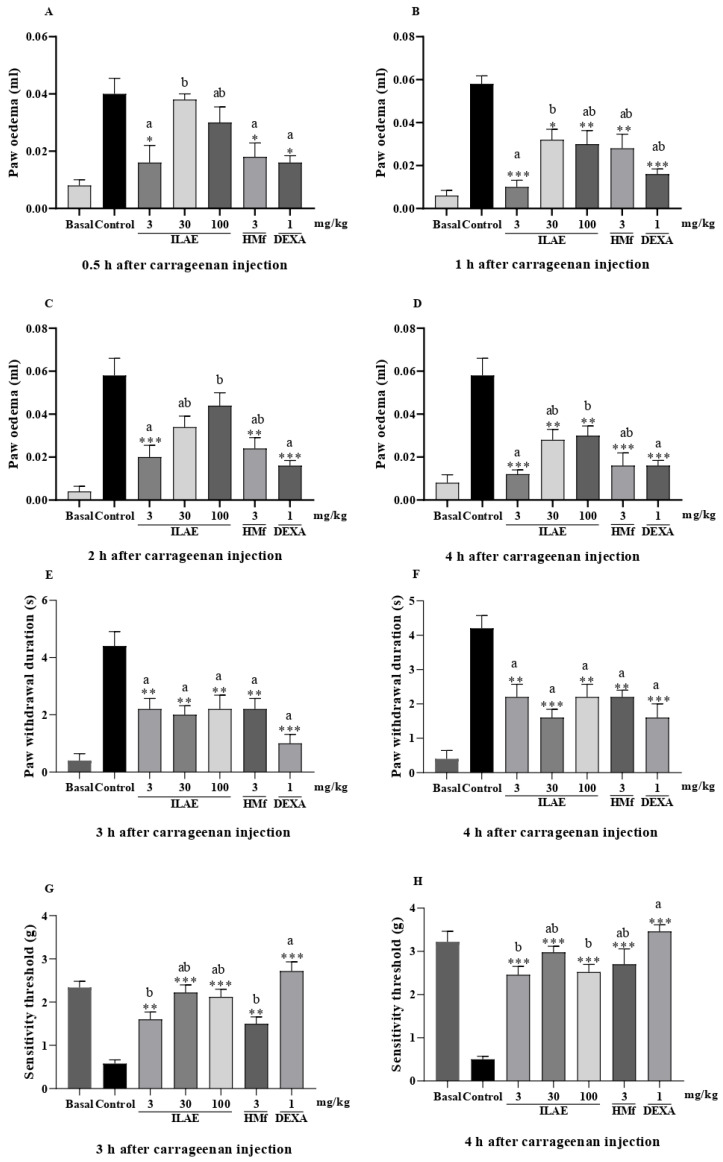
Effect of oral ILAE (3, 30 and 100 mg/kg), HMf (3 mg/kg), DEXA (1 mg/kg), or the vehicle (control) administration on (**A**–**D**) carrageenan-induced paw edema; (**E**,**F**) acetone-induced cold allodynia, and (**G**,**H**) mechanical hyperalgesia in mice. Each point represents the mean ± SEM of 5 animals. (*) symbol indicates the significant differences between treated groups compared with control group (* *p* < 0.05, ** *p* < 0.01 and *** *p* < 0.001). The letters (a and b) indicate the significant differences between treated groups (*p* < 0.05).

In the carrageenan-induced paw inflammation model, the oral exposure to ILAE (3 mg/kg), HMf (3 mg/kg) and DEXA (1 mg/kg) significantly reduced edema formation at 0.5 h, with reductions of 60.0%, 55.0%, and 60.0%, respectively, compared to the control (all with *p* < 0.05) (Figure 5A). A comparison among treatments showed that ILAE (3 mg/kg) and ILAE (30 mg/kg) (*p* < 0.05) were statistically different from each other (Figure 5A); DEXA (1 mg/kg) (*p* < 0.05) and ILAE (3 and 100 mg/kg) and HMf (3 mg/kg) were not statistically different (Figure 5A).

At 1 h after carrageenan injection (Figure 5B), ILAE (3 mg/kg) inhibited edema formation by 82.7% (*p* < 0.001), while HMf (3 mg/kg) inhibited edema formation by 51.7% (*p* < 0.01), and DEXA by 72.4% (*p* < 0.001) (all inhibitions were calculated relative to the control). When comparing the treatments, ILAE (3 mg/kg) and ILAE (30 mg/kg) (*p* < 0.05) showed a statistical difference (Figure 5B). At 2 h (Figure 5C), ILAE (3 mg/kg), HMf (3 mg/kg) and DEXA (1 mg/kg) significantly reduced edema formation by 65.5% (*p* < 0.001), 58.6% (*p* < 0.01) and 72.4% (*p* < 0.001), respectively, compared with the control. A statistical difference between ILAE (3 mg/kg) and ILAE (100 mg/kg) (*p* < 0.05) was observed throughout the evaluation (when compared to the treatments) (Figure 5C). In the final evaluation at 4 h (Figure 5D), all treatments showed a statistically significant effect (when compared to the control), highlighting ILAE (3 mg/kg), HMf (3 mg/kg), and DEXA (1 mg/kg), with 79.3% (*p* < 0.001), 72.4 (*p* < 0.001)%, and 72.4% (*p* < 0.001) inhibition of paw edema, respectively. No statistical differences were observed between ILAE (3 and 30 mg/kg), HMf (3 mg/kg) and DEXA (1 mg/kg) (Figure 5D).

In the carrageenan-induced paw edema model, the acetone-induced allodynia and oral administration of ILAE and HMf significantly reduced responses to cold stimuli, by 50.0% (ILAE 3 and 100 mg/kg, and HMf 3 mg/kg) and 54.0% (ILAE 30 mg/kg) at 3 h (Figure 5E), compared to the control group (all with *p* < 0.01). After 4 h (Figure 5F), the observed reduction was 47.6% (ILAE 3 and 100 mg/kg, and HMf 3 mg/kg) (*p* < 0.01) and 61.9% (ILAE 30 mg/kg) (*p* < 0.001), compared to control. Treatment with DEXA (1 mg/kg) showed a decrease of 77.2% at 3 h and 61.9% at 4 h (Figure 5E,F) compared to the control group (both with *p* < 0.001). No statistical differences were observed between all treatments (ILAE, HMf and DEXA), at 3 and 4 h (Figure 5E,F).

The results of mechanical hyperalgesia at 3 h after carrageenan paw injection (Figure 5G) showed that ILAE inhibited this parameter by 63.7% (3 mg/kg, *p* < 0.01), 73.8% (30 mg/kg, *p* < 0.001), and 72.6% (100 mg/kg, *p* < 0.001), while HMf (3 mg/kg, *p* < 0.01) inhibited by 61.3% compared to the control group. At 4 h (Figure 5H), ILAE treatments (3, 30 and 100 mg/kg) inhibited mechanical sensitivity by 79.6, 83.2, and 80.1%, respectively, and HMf (3 mg/kg) by 81.4%, all compared to the control group (*p* < 0.001). The positive control, DEXA, showed a significant reduction at all time points (78.6% after 3 h and 85.5% after 4 h), with *p* < 0.001, compared to the control group (Figure 5G,H). A comparison between the treatments demonstrated that DEXA (1 mg/kg) did not differ statistically from ILAE (30 and 100 mg/kg) at 3 h (Figure 5G); however, to 4 h no statistically differences were observed between DEXA (1 mg/kg), ILAE (30 mg/kg) and HMf (3 mg/kg) (Figure 5H).

**Figure 6:**
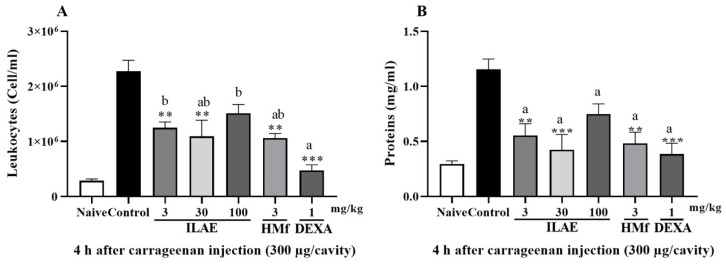
Effect of oral treatment with ILAE (3, 30 and 100 mg/kg), HMf (3 mg/kg), in carrageenan-induced pleurisy model, measured by (**A**) total leukocyte count and (**B**) protein dosage. The other induced groups received DEXA (1 mg/kg, p.o.), or the vehicle (control). The data are represented as the mean ± SEM of 5 animals. The (*) symbol indicates the significant differences between treated groups compared with control group (** *p* < 0.01 and *** *p* < 0.001). The letters (a and b) indicate the significant differences between treated groups (*p* < 0.05).

Oral treatment with ILAE (3 and 30 mg/kg) and HMf (3 mg/kg) significantly reduced leukocyte counts by 45.1%, 52.0%, and 53.4%, respectively, compared to control (*p* < 0.01) (Figure 6A). DEXA (1 mg/kg) decreased leukocyte migration by 79.2%, with *p* < 0.001 (Figure 6A). When compared to all treatments, DEXA (1 mg/kg) did not exhibit statistical differences from ILAE (30 mg/kg) and HMf (3 mg/kg) (Figure 6A); and there was also no statistical difference between the treatments obtained from *A. edulis* (ILAE and HMf) (Figure 6A). Protein exudation was significantly decreased by 63.3% (*p* < 0.001) in the ILAE (30 mg/kg) treatment, while ILAE (3 mg/kg) and HMf (3 mg/kg) decreased by 52.1 and 58.2%, respectively, compared to the control (*p* < 0.01) (Figure 6B). DEXA (1 mg/kg) decreased by 66.6% compared to the control (*p* < 0.001) (Figure 6B). No statistical differences were observed between all treatments (ILAE, HMf and DEXA) (Figure 6B).

**Figure 7:**
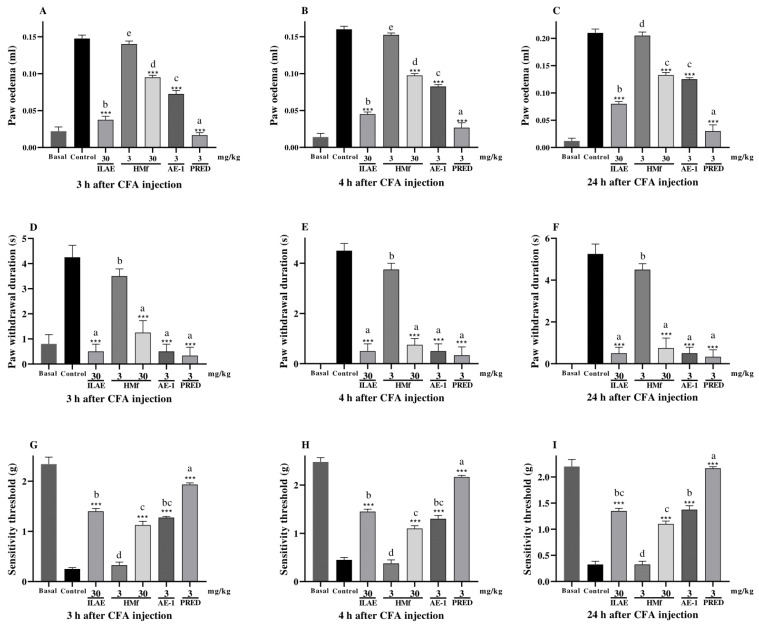
Effect of oral treatment with ILAE (30 mg/kg), HMf (3 and 30 mg/kg), AE-1 (3 mg/kg), PRED (3 mg/kg), or vehicle (control) on (**A**–**C**) CFA-induced paw edema; (**D**–**F**) acetone-induced cold allodynia, and (**G**–**I**) mechanical hyperalgesia in mice, by 3, 4 and 24 h after CFA injection. Each point represents the mean ± SEM of 5 animals. The (*) symbol indicates the significant differences between treated groups compared with control group (*** *p* < 0.001). The letters (a, b, c, d and e) indicate the significant differences between treated groups (*p* < 0.05).

When evaluating paw edema (Figure 7A–C), acetone-induced allodynia (Figure 7D–F) and mechanical hyperalgesia models (Figure 7G–I) influenced by CFA at different time intervals, all treatments showed significant inhibition compared to the control group (Figure 7A–F), except HMf (3 mg/kg) (Figure 7G–I). In the evaluation of CFA-induced paw edema, ILAE (30 mg/kg), HMf (30 mg/kg), and AE-1 (3 mg/kg) diminished in paw edema by 74.5%, 35.5%, and 50.8% at 3 h; 71.8%, 39.0%, and 48.4% at 4 h; and 61.9%, 36.9%, and 40.4% at 24 h, respectively, when compared with the control group (all with *p* < 0.001) (Figure 7A–C). PRED (3 mg/kg) showed a reduction of 89.1% at 3 h, 85.4% at 4 h, and 79.5% at 24 h compared to the control group (all *p* < 0.001) (Figure 7A–C). The comparison between all treatments showed statistical differences among them (*p* < 0.05) (Figure 7A,B), at 3 and 4 h; however, the remaining treatments HMf (30 mg/kg) and AE-1 (3 mg/kg) did not exhibit statistical differences among them at 24 h (Figure 7C).

When assessing acetone-induced allodynia, ILAE (30 mg/kg), HMf (30 mg/kg), and AE-1 (3 mg/kg) showed inhibition of acetone-induced allodynia by 88.2%, 70.5%, and 88.1% at 3 h; 88.8%, 83.3% and 88.7% at 4 h; and 90.4%, 85.7%, and 90.3% at 24 h, respectively, all with *p* < 0.001, when compared to the control group (Figure 7D–F). PRED (3 mg/kg) showed inhibition of 92.1% at 3 h, 92.6% at 4 h, and 93.6% at 24 h, when compared to the control, with *p* < 0.001 (Figure 7D–F). Among the treatments, a statistically significant difference (*p* < 0.05) was observed only for HMf (3 mg/kg) at 3, 4, and 24 h (Figure 7D–F).

In the mechanical hyperalgesia model following CFA injection, ILAE (30 mg/kg), HMf (30 mg/kg), and AE-1 (3 mg/kg) exhibited inhibitions of hyperalgesia by 82.1%, 77.7%, and 80.3% at 3 h; 68.9%, 59.0%, and 65.3% at 4 h; and 75.9%, 70.4%, and 76.3% at 24 h, respectively, all with *p* < 0.001, when compared to the control group (Figure 7G–I). PRED (3 mg/kg) demonstrated an inhibition of 87.0% at 3 h, 79.2% at 4 h, and 84.9% at 24 h, compared to the control, with *p* < 0.001 (Figure 7G–I). The comparison between treatments showed that PRED (3 mg/kg) (*p* < 0.05) exhibited statistically significant differences compared to all treatments at 3, 4 and 24 h (Figure 7G–I); however, the remaining treatments ILAE (30 mg/kg) and AE-1 (3 mg/kg) did not show statistical differences among themselves, maintaining their effect at 3, 4 and 24 h (Figure 7G–I).

## 3. Discussion

This study provides the first description of the secretory structures and their chemical components in *A. edulis* leaves. In addition, it reveals the acute anti-inflammatory and antinociceptive effects, as well as the prolonged anti-inflammatory benefits, resulting from the infusion of *A. edulis* leaves. Our results contribute significantly to the identification of the plant material and to the elucidation of the ethnobotanical use of this plant in traditional South American medicine for the treatment of inflammatory diseases [2].

*Allophylus edulis* is naturally distributed throughout South America in biomes such as the Amazon, Caatinga, Cerrado, Atlantic Forest, and Pantanal [19]. It is described as a tree with an erect trunk reaching up to 17 m in height [20], with trifoliate leaves (Figure 1b) that are chartaceous in consistency, with lateral leaflets smaller than the terminal leaflets [21]. Histological sections of the leaves revealed secretory structures, including ducts, glandular trichomes, laticifers, and idioblasts (Figure 1 and Figure 2). Some of these have been previously identified in *Allophylus* species and other genera within the Sapindaceae family [22,23,24]. Laticifers and idioblasts, identified in *A. sericeus* (Cambess.) Radlk., were stored in the pith and phloem [24]. In *A. edulis*, these structures, when treated with dyes, reveal the presence of essential oils, latex, phenolic compounds, tannins, and alkaloids (Figure 1 and Figure 2). The main components of latex in the Sapindaceae family are the lipid fraction with predominant compounds being terpenes (essential oils and resins), carbohydrates (mucilage), proteins, and phenolic compounds [24]. Furthermore, the detection of alkaloids (Figure 2e), even in a qualitative context, seems to be a novel observation for this species, as they have only been found in the latex of the genus *Paullinia* [24] and in the hydromethanolic extract of the leaves of *A. africanus* P. Beauv. [25]. Idioblasts were frequently found in the leaves of *A. edulis* and formed extensive rows, whereas laticifers formed less extensive rows and were less abundant.

Our research group investigated the presence of essential oils in *A. edulis* leaves [26]. This exploration is driven by the chemical diversity of terpenes, as well as their potential applications in pain and inflammation models. These studies [10,11,23] demonstrated promising anti-inflammatory activity, regardless of the specific terpene profile. The presence of phenolic and polyphenolic compounds in the leaves was revealed by partitioning of the leaf infusion (lyophilized) and spectrophotometric quantification of these secondary metabolites. Both the ILAE, EAf, and HMf showed a moderate concentration of total phenolics, flavonoids, flavonols, and condensed tannins (Table 1).

Our results showed higher concentrations of phenolic compounds and flavonoids compared to those reported by Tirloni et al. [7], who used an aqueous leaf extract obtained at a low temperature (4 °C). The higher yields observed in our study may be attributed to the use of infusion, as traditional extraction methods are known to enhance the release of total polyphenols at temperatures above 60 °C [27]. Histological analysis confirmed the presence of phenolic compounds in idioblasts located in the midrib (Figure 2c), petiole (Figure 2g), and petiolule (Figure 2k), supporting the anatomical distribution of these compounds in leaf tissues, but not directly related to the temperature-dependent extraction efficiency.

The purification of HMf resulted in AE-1. This derivative of vitexin has a α-rhamnosyl moiety linked at the C-2″ of the glycosidic unit. The LC-DAD analysis (Figure 4) confirmed that AE-1 is a notable constituint of the lyophilized infusion (ILAE) under the tested chromatographic conditions. The AE-1 has been previously identified in alcoholic extracts of *A. edulis*, showing both angiotensin-converting enzyme inhibitory [1] and hepatoprotective effects [8]. Aqueous extracts of *A. africanus*, with the presence of vitexin 2″-O-rhamnoside, also show anti-inflammatory potential [28]. Other species presenting this compound and its analogs have demonstrated potential antinociceptive [29], anti-inflammatory [30,31,32,33], antioxidant [34,35,36], and immunomodulatory effects [37]. In silico predictive methods represent an alternative approach to accelerate preclinical assessment of the potential adverse effects of compounds, thereby reducing the time, cost, and dependence on animal testing. ProTox-II is a computational tool that differs from other models by its classification, which divides the prediction scheme into different levels of toxicity. The current study revealed that in silico toxicity output predicted AE-1 to be a non-hepatotoxic, non-carcinogenic, non-mutagenic, and non-cytotoxic agent (Table 2). Finding such agents is crucial in various fields, especially pharmaceuticals, where safety is paramount. However, AE-1 has been predicted to be immunotoxic (Table 2). This prediction was made using a computational (in silico) model, meaning it has not yet been confirmed by laboratory or animal experiments. This result should therefore be interpreted with attention, particularly because the compound has also shown immunomodulatory and anti-inflammatory effects in previous studies [30,31,32,33,37].

The present study describes for the first time the anti-inflammatory effects of ILAE, in an attempt to provide evidence supporting the ethnobotanical use (anti-inflammatory effects) of the leaves of this species in Brazil [1,2,3]. Inflammation is one of the physiological responses associated with oxidative stress. Oxidative stress can activate signaling pathways that promote inflammation by stimulating the production of pro-inflammatory cytokines and chemokines, attracting immune cells to the site of injury or infection. Derivatives from natural sources with antioxidant properties, are studied for their potential to mitigate oxidative stress and inflammation, thereby potentially offering therapeutic benefits. In this context, we evaluated the antioxidant capacity of the leaf infusion (ILAE) and its fractions. The results revealed low IC_50_ values in the free radical scavenging assays (≤28.07 µg/mL, DPPH), with HMf standing out as the most potent fraction, exhibiting an IC50 of 15.17 µg/mL (DPPH) (Table 3).

Notably, these results are more promising than those previously reported for the aqueous leaf extract, which showed an IC_50_ of 45.8 µg/mL (DPPH) [7]. These findings suggest that the antioxidant activity observed in ILAE, and particularly in HMf, may be partially attributed to their high contents of total phenols, flavonoids, flavonols, and condensed tannins (Table 3). The antioxidant potential was also observed in the evaluation of the ethanolic [38] and methanolic extracts [39] of *A. edulis* fruits. This property is largely attributed to the presence of flavonoids, which have the ability to scavenge free radicals by forming less reactive phenoxyl flavonoid radicals due to their hydrogen atom donating ability [40]. The presence of flavonoids, such as vitexin derivatives, may also support the endogenous antioxidant defenses during a chronic inflammatory process [41].

To evaluate the anti-inflammatory effects of *A. edulis*, we used experimental models of carrageenan- and CFA-induced paw edema, cold allodynia, and mechanical hyperalgesia, and carrageenan-induced leukocyte migration in mice. These models were selected based on the methodology outlined by our research group [10,11,12], who studied the essential oil from the leaves of this species.

Initially, the acute anti-inflammatory effects of ILAE and HMf (phenolic compounds and flavonoid-rich fraction) were evaluated using carrageenan-induced inflammation models. Carrageenan, a mucopolysaccharide, triggers inflammation by activating genes for cytokines and promoting the migration of immune system cells [42]. The carrageenan-induced inflammatory response is a local process characterized by the cardinal signs typical of inflammation, including redness, heat, pain, and edema. These manifestations result from increased blood flow to the inflamed site, driven by changes in the local microvasculature, resulting in the extravasation of fluids, plasma proteins, and leukocytes, as well as pro-inflammatory cytokines [43]. The treatments ILAE (3 mg/kg) and HMf (3 mg/kg) obtained from *A. edulis* reduced paw edema development time (Figure 5A–D) during the early (up to 2 h) and late (3–4 h) phases of carrageenan-induced inflammation; these treatments prove to be as effective as dexamethasone (DEXA), at all evaluation times (Figure 5A–D). The three tested doses of ILAE did not exhibit a clear dose-dependent effect, suggesting that increasing the concentration did not proportionally enhance the observed response. This outcome is not unexpected when dealing with complex plant extracts rich in polyphenols, whose pharmacological activity may, in part, arise from the combined action of multiple constituents that interact synergistically, antagonistically, or influence each other’s stability and bioavailability. Therefore, the absence of a classical dose–response relationship may reflect the intrinsic complexity of the extract matrix rather than a lack of efficacy at higher doses.

Carrageenan-induced inflammation action produces inflammatory and painful mediators responsible for activating and sensitizing peripheral nociceptors, resulting in cold allodynia and mechanical hyperalgesia [44]. The pain (anti-hyperalgesic) effects of ILAE and HMf could be observed in carrageenan induced cold and mechanical hyperalgesia (Figure 5E–H). ILAE and HMf significantly reduced the duration of cold hypersensitivity at 3 and 4 h. Although no significant differences were observed between the treatments (Figure 5E,F), when compared individually to the control, only ILAE (30 mg/kg) showed a statistically more pronounced effect at 4 h (Figure 5F), suggesting a relatively greater efficacy at this time point. All treatments were as effective as dexamethasone (DEXA) at 3 and 4 (Figure 5E,F). The ILAE and HMf also significantly reduced mechanical sensitivity at 3 and 4 h after injection of the tested dose (Figure 5G–H).

Previous studies conducted by our research group demonstrated that the essential oil extracted from the leaves of *A. edulis* exhibited anti-inflammatory activity, including consistent reductions in edema formation and sensitivity to cold and mechanical stimuli [11]. Interestingly, in the present study, the plant maintained its anti-inflammatory properties when we used an infusion prepared from the leaves. Although volatile compounds were not analyzed in this study, the infusion method used may have allowed partial extraction of some essential oil by adding the plant material and letting the mixture rest covered for a few minutes. Thus, leaf infusion, rather than essential oil, may result in higher yields of products derived from *A. edulis* without compromising the plant’s anti-inflammatory effect. The essential oil yield from fresh leaves of *A. edulis* has been reported to range between 0.07% and 0.6% [26]. In contrast, the lyophilized infusion prepared in the present study, also derived from fresh leaves, yielded 4%, indicating a substantially higher extraction efficiency for water-soluble constituents.

After evaluation of the efficacy of *A. edulis* in reducing total leukocyte count (Figure 6A) and protein extravasation (Figure 6B) in carrageenan induced pleurisy model, the results indicated that the lowest doses of ILAE (3 and 30 mg/kg) and HMf (3 mg/kg) are effective. Although ILAE did not demonstrate a dose-dependent anti-inflammatory effects in acute assessments (paw edema and pleurisy), no significant differences were observed compared to the positive control, particularly at the end of the evaluation (4 h). These findings suggest a comparable profile to DEXA.

Although leukocyte migration is a critical mechanism of inflammatory response, chronic inflammation can exacerbate the intensity and duration of the process. The observed regulatory effect on leukocyte infiltration in the pleura may be attributed to the changes instigated by the flavonoids contained in the treatments, such as changes in leukocyte rolling ability, adhesion, and transmigration [45,46]. The presence of a 2, 3 double bond and the 4-keto group of the C ring, have been identified as key requirements for the inhibition of adhesion molecule expression [47]. This effect may be in line with the in vivo antioxidant capacity [37] and the protective effect on endothelial cells and injured cardiac myocytes [48], described for the vitexin 2″-O-rhamnoside.

Oral treatment with ILAE, HMf and AE-1 was evaluated in the CFA-induced (3, 4 and 24 h) paw edema, mechanical hyperalgesia, and cold sensitivity (Figure 7A–I). The CFA-induced paw edema is recognized as a model of chronic or persistent inflammation resulting from the continuous release of antibodies stimulating phagocytosis, cytokine secretion by mononuclear phagocytes, and the expression of costimulators for T cell activation and proliferation [49]. To investigate the potential persistent effects, the intermediate dose of ILAE (30 mg/kg) was selected for the CFA-induced inflammation model, as it consistently produced effects comparable to both the lower (3 mg/kg) and higher (100 mg/kg) doses across the different phases of inflammation in the carrageenan models, where no clear dose–response relationship was observed. Given that only a low dose of 3 mg/kg was previously tested, a higher dose of 30 mg/kg was introduced to evaluate the persistence of HMf effects during prolonged inflammation. Furthermore, AE-1 derived from *A. edulis*, was tested to explore any potential correlation between its presence and the observed pharmacological effects. The oral treatment of ILAE (30 mg/kg), HMf (30 mg/kg), and AE-1 (3 mg/kg) observed at 3, 4, and 24 h post-CFA injection, showed a significant effect in three analyzed parameters (such as edema, cold allodynia and mechanical sensitivity) (Figure 7A–I); however, the pretreatment with HMf (3 mg/kg) did not inhibit the formation of edema, duration of cold and reduced mechanical sensitivity at all assessment times (Figure 7A–I). Notably, ILAE (30 mg/kg), HMf (30 mg/kg), and AE-1 (3 mg/kg) demonstrated comparable efficacy to PRED (3 mg/kg), as they attenuated the duration of cold sensitivity in intervals of 3, 4 and 24 h intervals, demonstrating its anti-hyperalgesic potential (Figure 7D–F). These results are consistent compared to a previous study conducted by our research group with the essential oil of *A. edulis* (30 mg/kg) observed after 1, 3, 6, 9, and 12 days after CFA injection. Meanwhile, the reduction in mechanical hyperalgesia remained virtually unchanged until the twelfth day, and the decrease in cold-induced allodynia exhibited a transient effect [11]. Thus, *A. edulis* can reduce the persistent effect induced by CFA, on the evaluated parameters. Further studies are warranted to investigate the underlying mechanisms of its anti-inflammatory activity, including detailed biochemical analyses.

Although our study provides valuable insights, it has some limitations that should be highlighted. The relatively small number of animals used, as well as the limited range of doses tested for HMf and AE-1 in the experimental models, was due to ethical restrictions regarding animal use. Furthermore, although anti-inflammatory effects were confirmed in the CFA model, evaluations were limited to 24 h, whereas in the study by Santos et al. [11], the essential oil from *A. edulis* was reported to exhibit sustained activity over several days. Further studies are needed to explore additional properties of AE-1 and to more precisely elucidate its mechanism of action. Since this study was conducted in an animal model, the validation of the therapeutic use of *A. edulis* leaves for treating inflammatory conditions in humans remains limited. The anti-inflammatory and analgesic effects of flavonoid-rich samples are noteworthy [50]. While flavonoids alone can reduce inflammation pain [51], their beneficial effects on the inflammatory agents such as carrageenan and CFA may be related not only to the direct reduction in inflammatory factors or oxidative enzymes but also to changes in amino acid metabolism, which is crucial for pain transmission [52]. This includes inhibition of regulatory enzymes, antioxidant properties, influence on arachidonic acid metabolism, and genetic and cellular modulation [53,54,55].

## 4. Materials and Methods

### 4.1. Drugs and Solvents

λ-Carrageenan, Complete Freund’s Adjuvant (CFA), prednisolone (PRED), Bradford reagent, quercetin hydrate, catechin hydrate, gallic acid, 2,2-Diphenyl-1-picrylhydrazyl (DPPH), 2,2′-azino-bis(3-ethylbenzothiazoline-6-sulfonic acid) diammonium salt (ABTS), butylated hydroxytoluene (BHT), lipophilic Sephadex resin (Sephadex LH-20), linoleic acid ≥ 98%, β-carotene, Tween 40, syringic acid, rutin and potassium persulfate were purchased from Sigma-Aldrich (St. Louis, MO, USA). TCL Silica gel 60 was purchased from Merck KGaA (Darmstadt, Germany). Silica gel 60 GF254 was purchased from Merck/Supelco, Darmstadt, Germany). Dexamethasone (DEXA) was purchased from EMS (Hortolândia, SP, Brazil). L-Ascorbic acid from Dinâmica (São Paulo, SP, Brazil). Turk’s solution from Newprov (Pinhais, PR, Brazil). Methanol and *n*-hexane from Neon (Suzano, SP, Brazil). Ethyl acetate from Proquimios (Rio de Janeiro, RJ, Brazil). CD_3_OD used on Nuclear Magnetic Resonance (NMR) was LC grade and purchased from Cambridge Isotope Laboratories (Andover, MA, USA). All other chemicals, including reference compounds and reagents, were of analytical grade.

### 4.2. Plant Material

The *A. edulis* leaves were collected at the Medicinal Plant Garden of the Federal University of Grande Dourados (UFGD), in the city of Dourados (Mato Grosso do Sul, Brazil, 22°11′43.7″ S 54°56′08.5″ W), identified by Dr. Zefa Valdivina Pereira, and deposited in the UFGD herbarium (code 6283). The authorization for access to the Brazilian genetic heritage was obtained from the National System for the Genetic Heritage and Associated Traditional Knowledge Management (SisGen-A51F665).

### 4.3. Histochemical Analyses

Freehand sections of fresh leaves of *A. edulis* were cut transversely sectioned and, subsequently submitted to different treatments to investigate the chemical composition of the secretions present in the glandular trichomes, ducts, laticifers and idioblasts. Various reagents were used for the detection of classes of compounds and the description of reagents/compounds are as follows: (a) Sudan III to verify the presence of lipophilic compounds [56]; (b) Nadi reagent to detect terpenoids [57]; (c) ferric chloride [58] and potassium dichromate [59] to reveal the presence of phenolic compounds; (d) vanillin—hydrochloric acid to evidence tannins [60]; (e) Dragendorff’s reagent [61] to detect the presence of alkaloids and (f) oil red O reagent to detect latex [62]. Untreated sections were used as controls. Slides were observed under bright field and the photomicrographs were obtained using an Olympus CX31 (Olympus, Kyoto, Japan) attached to a C7070 control unit.

### 4.4. Infusion Preparation, Fractioning, Isolation, and NMR Analysis

The infusion of *A. edulis* leaves (ILAE) was obtained by boiling distilled water (10 L), which was then placed over fresh chopped *A. edulis* leaves (1.0 kg). After infusing for 15–25 min, it was filtered and lyophilized (Lyophilizer Christ, Osterode am Harz, Germany). The lyophilized infusion (ILAE, 40 g) was stored in a freezer at −5 °C until needed for the experiments.

Part of the ILAE (30 g) was dissolved in MeOH/H_2_O (1:1) and partitioned with *n*-hexane and ethyl acetate, to obtain the *n*-hexane (Hf, 4.6 g), ethyl acetate (EAf, 10.5 g) and hydromethanol (HMf, 14.7 g) fractions, which were subsequently analyzed by Thin-layer chromatography (TLC) plates (silica gel 60 or GF254), accomplished by UV irradiation at 254 and 366 nm, and/or by spraying with a H_2_SO_4_/MeOH (1:1), H_2_SO_4_/anisaldehyde/acetic acid (1:0.5:50 mL) solutions followed by heating at 100 °C or Dragendorff’s solution. The HMf obtained from partitioning was subjected to column chromatography on Sephadex LH-20 eluted sequentially with H_2_O, H_2_O/MeOH mixtures at ratios of 8:2, 6:4, 4:6, and 2:8, followed by pure MeOH, resulting in sub-fractions HMf-1 to HMf-37. Sub-fraction HMf-14 was further purified by preparative TLC, eluted with CHCl_3_/MeOH (8:2), yielding a compound designated as AE-1 (160 mg).

The ^1^H and ^13^C NMR (300 and 75.5 MHz) spectra were recorded on a Bruker Ascend 300 spectrometer (Bruker, Berlin, Germany), in ppm, using CD_3_OD as solvent.

### 4.5. Liquid Chromatographic Analysis of ILAE

The analysis of ILAE was performed on a liquid chromatograph equipped with a diode array detector (DAD) (Shimadzu, Kyoto, Japan), using a spectral range of 200 to 800 nm. The system consisted of a binary pump LC-20AD, an automatic sampler SIL-20A HT, a column oven CTO-20A, a DAD SPD-M20A, and a C18 column (2 mm × 75 mm) with 0.22 µm particles. A gradient elution system was used, with mobile phase A consisting of 0.1% formic acid and solvent B being methanol. The percentage of solvent B started at 0%, increased from 0 to 30% over 14 min, then from 30 to 100% over 10 min, decreased from 100 to 0% over 4 min, and was held at this proportion for 2 min. The column oven was maintained at 30 °C during the analysis, with a flow rate of 0.45 mL/min. A 5 µL aliquot of each sample, at a concentration of 1 mg/mL and previously filtered through a 0.22 µm analytical filter, was injected. Standards (catechin, syringic acid and rutin) and isolated compound AE-1 were quantified by external calibration at different concentrations.

### 4.6. Quantification of Constituents: Total Phenols, Flavonoids, Flavonols, and Condensed Tannins

Solutions of ILAE, EAf, and HMf were individually prepared in methanol at concentrations appropriate for each assay. All assays were performed in triplicate, and absorbance readings were taken using a UV-Vis spectrophotometer (Bel Photonics, Monza, Italy). (A) total phenols were quantified using the Folin–Ciocalteu method. A 200 μL aliquot of the 1 mg/mL sample solution was mixed with 1.0 mL of distilled water and 0.5 mL of Folin-ciocaleu’s reagent (1:10 *v*/*v*). After mixing, 1.5 mL of 2% aqueous sodium bicarbonate were added, and the mixture was allowed to stand for 30 min with intermittent shaking. Absorbance was read at 760 nm [63,64]. A calibration curve was constructed using gallic acid (10–100 μg/mL) expressed as: y = 1.3516x + 0.1098 (R^2^ = 0.9802). (B) flavonoids were determined by mixing 500 μL of the 2 mg/mL sample with 1.5 mL of methanol, 100 μL of 10% aluminum chloride (AlCl_3_), 100 μL of 1 M potassium acetate, and 2.8 mL of distilled water. After incubation at room temperature for 30 min, absorbance was measured at 415 nm [63,64]. A calibration curve with quercetin (10–100 μg/mL) was expressed as: y = 12.94x − 0.0148 (R^2^ = 0.9991). (C) Flavonols were quantified by mixing 2.0 mL of the 2 mg/mL sample with 2.0 mL of 2% AlCl_3_ (in ethanol) and 3.0 mL of 50 g/L sodium acetate. The mixture was incubated for 2.5 h at 20 °C, and the absorbance was read at 440 nm [63]. Quercetin (10–100 μg/mL) was used as the standard, with a calibration curve: y = 26.143x + 0.3571 (R^2^ = 0.9885). (D) condensed tannins were analyzed using the vanillin–HCl method. A 500 μL aliquot of the 10 mg/mL sample solution was mixed with 3.0 mL of 4% vanillin in methanol and 1.5 mL of concentrated HCl. The solution was incubated at room temperature for 15 min before reading at 500 nm [63,64]. A catechin calibration curve (0.02–0.2 mg/mL) was used: y = 1.5666x − 0.0412 (R^2^ = 0.9905).

In all cases, results were expressed as mg of standard equivalent per gram of dry extract: gallic acid (GAE), quercetin (QE), or catechin (CE), depending on the assay.

### 4.7. In Silico Bioactivity and Toxicity Prediction of Vitexin 2″-O-Rhamnoside

The canonical Simplified Molecular Input Line Entry System (SMILES): [CC5OC(OC1C(O)C(O)C(CO)OC1c3c(O)cc(O)c4c(=O)cc(c2ccc(O)cc2)oc34)C(O)C(O)C5O] of vitexin 2″-O-rhamnoside was used as input to generate in silico toxicity predictions on the ProTox II server (https://tox-new.charite.de/protox_II, accessed on 12 January 2025), and the LD_50_ values were estimated according to the literature [65].

### 4.8. Antioxidant Activity

#### 4.8.1. Radical Scavenging Activity

The ABTS radical scavenging method [63,66], involved generating the ABTS radical by mixing ABTS (7.0 mM) and potassium persulfate (140 mM) and allowing it to sit in the dark for 16 h at room temperature. The resulting ABTS+ solution was then diluted with ethanol (P.A.) to obtain an absorbance of 0.700 ± 0.05 at 734 nm. Different concentrations of ILAE, EAf, and HMf (0.6–0.01 mg/mL in methanol) were added to this solution, and the absorbances were measured after 6 min. BHT was used as a positive control. An ABTS solution without the addition of samples was used as a control. The ABTS+ scavenging activity was calculated as ABTS+ scavenging activity (%) = (Abs Control − Abs Sample/Abs Control) × 100. The results were expressed as IC_50_.

The DPPH scavenging method [63,67] used different concentrations (0.6–0.01 mg/mL in methanol) of ILAE, EAf and HMf mixed with DPPH (0.1 mM). After incubating for 30 min in the dark at room temperature, the absorbance was measured at 515 nm using a spectrophotometer (Bel Photonics, Monza, Italy). The experiments were conducted in triplicate and BHT was used as a positive control. A DPPH solution without the addition of samples was used as a control. The percentage of DPPH inhibition was calculated as follows: I% = (Abs Control − Abs Sample/Abs Control) × 100. The results were reported as IC_50_.

#### 4.8.2. Lipid Peroxidation Assay

The antioxidant activity of ILAE, EAf and HMf was evaluated using the β-carotene/linoleic acid method [63,68]. A solution of β-carotene was prepared (2 mg/mL of β-carotene in chloroform mixed with 20 μL of 99% linoleic acid and 200 μL of Tween 40). After removal of chloroform, an emulsion was formed by vigorously stirring the solution with oxygen-rich distilled water. An aliquot of 2.5 mL of the emulsion was then mixed with the samples at various concentrations (0.01–1 mg/mL). Absorbance at 470 nm was measured immediately after preparation. The solutions were then placed in a 50 °C water bath, and absorbance readings were taken every 20 min to follow oxidation until the β-carotene coloration disappeared within 100 min. Antioxidant activity, measured as the percentage of bleaching inhibition, was determined using the formula %AA = 100 − [(Ai − At)/(A’i − A’t) × 100]. Ai = initial absorbance of the sample, At = after 100 min of incubation at 50 °C, A’i = initial absorbance of the control, and A’t = control after 100 min of incubation at 50 °C. The results were reported as IC_50_. The assay was performed in triplicate.

### 4.9. Pharmacology Studies

#### 4.9.1. Animals and Ethical Clearance

Experiments were performed on male and female Swiss mice (25–30 g), from the central animal house facility of the Federal University of Grande Dourados. Animals were housed in polypropylene cages measuring 30 cm (length) × 20 cm (width) × 13 cm (height) at 22 ± 2 °C with a 12:12 h light-dark cycle, with free access to commercial pelleted food and water. All experimental procedures were in accordance with the Ethical Committee in Animal Experimentation of UFGD (n. 05.2021).

Male mice were used for the carrageenan- and CFA-induced paw inflammation models, while females were used in the pleurisy assay due to their typically stronger inflammatory response in this model, characterized by enhanced leukocyte recruitment, an effect likely influenced by estrogenic modulation. Although sex differences in inflammatory responses are acknowledged and guided the selection of sexes for each model, the investigation of these differences was beyond the scope of the present study. Animals were randomly assigned to groups.

To evaluate dose–response effects, three doses of ILAE (3, 30, and 100 mg/kg, orally) were tested, based on previous studies from our research group that investigated the anti-inflammatory activity of *A. edulis* essential oil [10,11,12].

The ILAE, HMf, and AE-1 were solubilized in 0.9% saline solution and administered to the animals according to their body weight.

#### 4.9.2. Paw Inflammatory Model Induced by Carrageenan

Male Swiss mice (n = 5) were distributed in groups according to oral (gavage) treatments. Groups receiving different doses of ILAE (3, 30 or 100 mg/kg), HMf (3 mg/kg), DEXA (1 mg/kg) or control (0.9% saline solution) received the oral treatment. After 1 h, the inflammation was induced by injection of carrageenan (300 μg/paw, 50 μL in sterile 0.9% saline) into the right paw and 50 μL of 0.9% saline solution into the contralateral paw to serve as a control [11,69]. The basal group (physiological control) received no treatment or injections. Edema was measured at 0.5, 1, 2 and 4 h using a paw plethysmometer (PANLAB Harvard). In the same experiment, the animals were subjected to cold allodynia using acetone [70], and mechanical hyperalgesia (Von Frey test) assessed using an electronic von Frey apparatus [71]. Both parameters were measured 3 and 4 h after carrageenan injection.

#### 4.9.3. Pleurisy Induced by Carrageenan

Different groups of female Swiss mice (n = 5) were treated orally with different doses of ILAE (3, 30 or 100 mg/kg), HMf (3 mg/kg), DEXA (1 mg/kg) or control (0.9% saline solution). The naive group was treated orally and received intrapleural injection of sterile saline solution (0.9%). Inflammation of the pleura (pleurisy) was induced by application of 100 μL of 1% carrageenan into the pleural cavity of mice [11,72]. The euthanasia protocol (ketamine—150 mg/kg, intraperitoneal (i.p.) + xylazine—15 mg/kg, i.p.) was conducted 4 h after carrageenan, then a wash was obtained after introducing 1 mL of phosphate-buffered saline (PBS) into the thoracic cavity and the pleural exudate was collected. The exudate volume was measured, 20 μL was diluted in Turk’s Liquid (1:20) and used to determine the total number of leukocytes present in a Neubauer chamber. To evaluate protein extravasation, a portion of the exudates were centrifuged, and the protein concentrations were determined by the Bradford method [73].

#### 4.9.4. Inflammatory Paw Model Induced by CFA

Male Swiss mice (n = 5) were treated orally with ILAE (30 mg/kg), HMf (3 and 30 mg/kg), AE-1 (3 mg/kg), PRED (3 mg/kg) or sterile saline solution 0.9% (control). The basal group (physiological control) received no treatment or injections. Inflammation was induced by injection of a suspension of CFA (20 μL/right paw) and 0.9% saline solution (20 μL) in the contralateral paw [74]. Edema, cold sensitivity, and mechanical sensitivity were measured 3, 4, and 24 h after CFA injection, and the methodology used is described above.

### 4.10. Statistical Analysis

Statistical comparisons were performed using a one-way analysis of variance (ANOVA) followed by Tukey’s test, and the differences were considered statistically significant when *p* < 0.05. The percentage of inhibition was calculated by the control group. All statistical calculations and graphs were prepared using GraphPad Prism version 8.0 for Windows (GraphPad Software, San Diego, CA, USA). The IC_50_ was plotted in a graph of I% versus sample concentration.

## 5. Conclusions

The present study demonstrates the chemical diversity of the secretory structures of *A. edulis* leaves and highlights the prevalence of polyphenolic substances in the leaf infusion. The infusion (ILAE) and its fraction (HMf) exhibited strong antioxidant activity in vitro, as well as significant anti-inflammatory and antihyperalgesic effects in vivo. AE-1 shows anti-inflammatory effects. These effects may contribute to explaining the popular use of *A. edulis* (Sapindaceae) leaves in the treatment of inflammatory conditions.

## Figures and Tables

**Table 1:** Total phenols, flavonoids, flavonols and condensed tannins of *A. edulis*.

Metabolites	ILAE	EAf	HMf
Total phenols (mg GAE/g)	177.56 ± 6.16	155.92 ± 4.59	159.56 ± 4.74
Flavonoids (mg QE/g)	64.29 ± 0.18	46.37 ± 0.18	63.10 ± 0.20
Flavonols (mg QE/g)	55.22 ± 0.11	23.71 ± 0.09	49.63 ± 0.42
Condensed tannins (mg CE/g)	43.78 ± 1.12	37.53 ± 1.31	44.88 ± 1.50

Values represent the mean of three measurements ± standard deviation.

**Table 2:** In silico toxicity prediction output of AE-1 in ProTox toxicity model.

Toxicity			
Target		Prediction	Probability
Hepatotoxicity		Inactive	0.81 ^a^
Carcinogenicity		Inactive	0.90 ^a^
Immunotoxicity		Active	0.98 ^a^
Mutagenicity		Inactive	0.73 ^a^
Cytotoxicity		Inactive	0.66 ^a^
Cytochrome inhibitors	CYP1A2	Inactive	0.99 ^b^
	CYP2C19	Inactive	0.99 ^b^
	CYP2C9	Inactive	0.90 ^b^
	CYP2D6	Inactive	0.95 ^b^
	CYP3A4	Inactive	0.99 ^b^
	CYP2E1	Inactive	0.98 ^b^
LD_50_ (mg/kg)		5000 (Class 5)	67.38 ^c^

^a^ Probability (scale from 0 to 1); ^b^ Probability of the compound inhibiting the isoforms (scale from 0 to 1); ^c^ Prediction accuracy (%).

**Table 3:** Antioxidant activity of lyophilized infusion (ILAE) and fractions (EAf and HMf) of *A. edulis* leaves.

Assays	ILAE	EAf	HMf	BHT
	IC_50_ (µg/mL)			
DPPH	27.88 ± 0.002	28.07 ± 0.003	15.17 ± 0.004	9.89 ± 0.002
ABTS	40.55 ± 0.029	61.80 ± 0.013	25.15 ± 0.039	9.75 ± 0.004
β-carotene/linoleic acid	117.9 ± 0.50	195.60 ± 0.09	55.44 ± 0.72	13.03 ± 0.04

The values represent the means of three measurements ± standard deviation.

## Data Availability

Data presented in this study is contained within the article. Further inquiries can be directed to the corresponding author.

## Abbreviations

The following abbreviations are used in this manuscript:

ABTS	2,2′-Azino-bis(3-ethylbenzothiazoline-6-sulfonic acid) diammonium salt
BHT	Butylated hydroxytoluene
CE	Catechin equivalent
CFA	Complete Freund’s Adjuvant
DEXA	Dexamethasone
DPPH	2,2-Diphenyl-1-picrylhydrazyl
EAf	Ethyl acetate fraction
GAE	Gallic acid equivalent
Hf	*n*-Hexane fraction
HMf	Hydromethanol fraction
ILAE	Lyophilized infusion of *A. edulis*
NMR	Nuclear Magnetic Resonance
PBS	Phosphate-buffered saline
PRED	Prednisolone
QE	Quercetin equivalent
SisGen	National System for the Genetic Heritage and Associated Traditional Knowledge Management
TLC	Thin-layer chromatography
UFGD	Federal University of Grande Dourados
